# An Integrative Approach to the Study of Filamentous Oligomeric Assemblies, with Application to RecA

**DOI:** 10.1371/journal.pone.0116414

**Published:** 2015-03-18

**Authors:** Benjamin Boyer, Johann Ezelin, Pierre Poulain, Adrien Saladin, Martin Zacharias, Charles H. Robert, Chantal Prévost

**Affiliations:** 1 Laboratoire de Biochimie Théorique, CNRS, UPR 9080, Univ Paris Diderot, Sorbonne Paris Cité, 13 rue Pierre et Marie Curie, 75005 Paris, France; 2 DSIMB team, Inserm UMR-S 665 and Univ. Paris Diderot, Sorbonne Paris Cité, INTS, 6 rue Alexandre Cabanel, 75015 Paris, France; 3 Ets Poulain, Pointe-Noire, Republic of Congo; 4 MTI, INSERM UMR-M 973, Université Paris Diderot-Paris 7, Bât Lamarck, 35 rue Hélène Brion, 75205 Paris Cedex 13, France; 5 Technische Universität München, Physik-Department, James-Franck-Str. 1, 85748 Garching, Germany; University of Copenhagen, DENMARK

## Abstract

Oligomeric macromolecules in the cell self-organize into a wide variety of geometrical motifs such as helices, rings or linear filaments. The recombinase proteins involved in homologous recombination present many such assembly motifs. Here, we examine in particular the polymorphic characteristics of RecA, the most studied member of the recombinase family, using an integrative approach that relates local modes of monomer/monomer association to the global architecture of their screw-type organization. In our approach, local modes of association are sampled via docking or Monte Carlo simulations. This enables shedding new light on fiber morphologies that may be adopted by the RecA protein. Two distinct RecA helical morphologies, the so-called “extended” and “compressed” forms, are known to play a role in homologous recombination. We investigate the variability within each form in terms of helical parameters and steric accessibility. We also address possible helical discontinuities in RecA filaments due to multiple monomer-monomer association modes. By relating local interface organization to global filament morphology, the strategies developed here to study RecA self-assembly are particularly well suited to other DNA-binding proteins and to filamentous protein assemblies in general.

## Introduction

The organization of biological objects as multimers, and specifically as symmetric multimers, is the norm rather than the exception in cells. In an instructive review [1], Goodsell and Olson listed possible ways that proteins self-organize in cells and suggested why such association modes provide favorable options for proteins to exert their function.

Among the possible organizations, helical symmetries are particularly well represented. This type of organization, which incorporates the characteristics of a rigid body displacement, can be described as a rotation of the body around a particular axis combined with a translation along this axis, and is referred to as a screw transformation [2]. When repeatedly applied to positioning a monomer with respect to the preceding one, screw transformations produce helices, as well as cyclic assemblies or linear arrangements in case the translation or the rotation, respectively, is null. The possibility of helical organizations arose naturally in the search for regular structures in proteins, and led Linus Pauling in 1951 to predict alpha helix [3] or quasi-straight segments such as *β*-sheets [4]. Soon after, the helical structures of DNA in the A, B or Z forms provided additional examples [5, 6].

Beyond the level of secondary structure, screw organization is widely encountered in the world of homo-oligomeric or polymeric protein association, where copies of the same protein assemble in organized quaternary structures that can attain impressive sizes. Recombinase proteins are a particularly relevant example. These proteins are involved in homologous recombination (HR) [7], where they catalyze the faithful repair of DNA double strand breaks in a process that is common to all realms of life [8]. For this purpose they interact with DNA molecules, either in the form of cyclic assemblies [9, 10] or as long filaments [11]. They often show a simpler organization than many fiber-forming proteins, as here the monomeric units tend to assemble into filaments that grow using only a single (*i*, *i* + 1) monomer interface. Beyond recombination, such organization is found in other DNA processing systems such as replication (DnaA) and in the protofilament building blocks of cytoskeleton fibers. In the recombinases, right-handed helical organization reflects the secondary structure of associated DNA, which is stretched by 50% [12] and unwound by 40% [13] with respect to standard B-DNA. Observed pitch variations between filaments of RecA-ATP (often called “active”, or “extended” form) and RecA-ADP (so-called “inactive”, or “compressed” form) have fueled the debate on the role of DNA stretching in the HR mechanism [14–16]. DNA-free forms of association have also been observed, involving dimers or hexameric rings for the prokaryotic RecA [17, 18], octameric rings for eukaryotic recombinases Rad51 or Dmc1 [9, 10] or even left-handed helices for the yeast RadA [19]. These observations were obtained using a panoply of methods including electron microscopy (EM) [17, 20]), atomic force microscopy (AFM) [10, 18] or X-ray crystallography [19, 21–23].

Attempts have been made to establish a relationship between these diverse forms of RecA association. Long considered unlikely [24], interconversion between the extended and compressed forms of the RecA filament has been demonstrated in recent years [25, 26]. In the same way, identification of two possible DNA-binding forms for RadA and Dmc1 as stacked octamers or as helical filaments [9, 10] raises the question of a possible interconversion between them. More generally, the passage between different forms of recombinase filaments has been proposed to play a role in the HR mechanism of DNA strand exchange [27, 28].

However, the problem of interconversion presupposes an understanding of the basic determinants of filament formation. This is not a straightforward undertaking. In principle, known crystal structures of the protein components should help us model the structures of the different assemblies that have been either proposed or observed at low resolution. Multidocking methods have been developed to treat just this type of problem. The general problem is highly complex, as supramolecular assemblies may be characterized by multiple, simultaneous component interactions, i.e. in which each component presents different interfaces with different partners. Further, their association may lead to a quaternary structure of fixed size, or else to an extended form as seen for example in bundled actin protofilaments. Complex assemblies of the first type, which may or may not be symmetrical, have been approached in several studies [29–33]. In these cases the overall architecture is highly constrained by steric requirements of the multiple interfaces whose resolution requires intensive combinatorial search. The case of homo-oligomeric structures has been approached via specific multiple docking methods [34–39]. These predictions generally took advantage of known C_*n*_ or D_*n*_ symmetry, either directly, by applying symmetry conditions during the conformational search (thus reducing the number of degrees of freedom searched), or indirectly, by filtering out the results that fulfilled the desired symmetry criteria. Finally, it must be kept in mind that protein monomers are, in general, flexible. When monomers undergo significant conformational changes, predicting their different modes of interaction remains a formidable challenge [40–42]. In such cases, characterizing the flexibility of the unbound proteins can help in limiting the space of possible interaction geometries [43, 44].

In line with early work of Eisenstein and coll. [34] and the recent study of Nivaskumar and coll. [45], we address here the question of fiber assembly from the point of view of two-component modes of interaction. We note in particular that each favorable monomer-monomer binding geometry defines a unique helical or cyclic organization, or mode of self-assembly. Such filaments may demonstrate considerable diversity, in which each distinct interface geometry gives rise to a structural family. Within a family, slight modifications of the interface can result in significant changes at the level of the overall filament morphology. Further, different interface geometries may be found in different regions of a filament.

The approach we present integrates interface sampling and filament construction using screw transformations to characterize putative binding modes. This makes use of a set of recently developed computational tools, called Heligeom, which aim at characterizing, manipulating and assembling structural units with a screw organization, and in which the structural units may be individual proteins or protein hetero-multimers (Boyer et al., in preparation). Heligeom relies on the structures of monomer-monomer interfaces both for deriving the transformations and for filament construction; for the latter it is thus complementary to other packages that apply known space group symmetries to obtain the structures of supra-assemblies (see for example the web servers PQS [46], PITA [47] and PISA [48]). Because Heligeom is bundled with the freely available Python/C++ library PTools [31, 49], our approach can therefore benefit from existing PTools functionalities such as coarse graining, energy calculations, and diverse sampling protocols, which can be combined to arrive at novel strategies for investigating helical assemblies.

Although more complex systems may also be treated using these tools, our specific targets in this article are recombinase protein assemblies, principally RecA, in which multiple association states have been documented. As will be seen, the RecA recombinase may be decomposed into two regions: a rigid core domain and more mobile regions. This decomposition allows an indirect treatment of the latter’s role. We show that this approach is well adapted for modelling a variety of fiber morphologies that correspond remarkably well to both known and proposed forms of recombinase protein assemblies.

## Methods

### Overall approach

**Fig 1:**
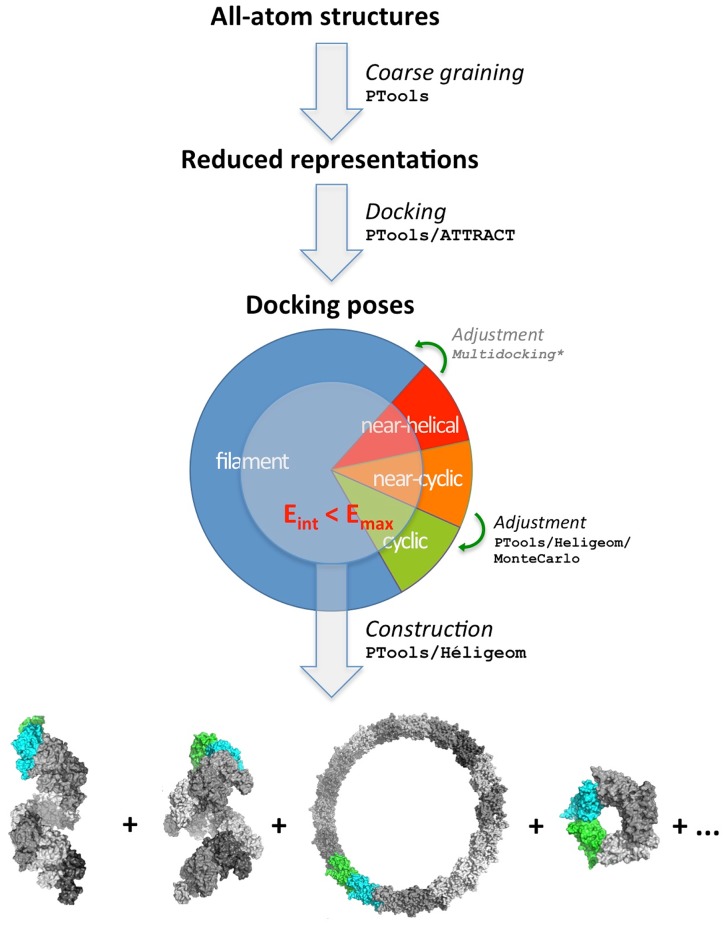
Overview. Binding geometries generated by a PTools/ATTRACT coarse-grained docking simulation are analyzed with Heligeom in terms of the helical parameters of regular assemblies that they define. The results are filtered based on relative energies and geometry considerations (see S1 Fig, supporting information). Binding geometries with near-cyclic organizations but suffering from steric clashes are submitted to an optimization process in which they are adjusted towards the two closest cyclic geometries (S1 Protocol, supporting information). Binding geometries leading to steric clashes that are also not in the near-cyclic category are currently not analyzed further but may be adjusted to helical organization in future developments. Heligeom can be applied to any of the final structures to construct filamentous or cyclic assemblies of arbitrary size for further analysis.

The approach we present combines the PyATTRACT and Heligeom modules of the PTools Python/C++ library [31], along with other functions of the library, in order to investigate the geometry of open oligomeric filaments (a schematic view of the overall approach is presented in Fig. 1). The process starts with the structure of a unique monomer of the system under study, which is reduced to coarse grain representation and then docked against itself using ATTRACT [49, 50]. The docking results are then processed by Heligeom to provide the helical parameters corresponding to regular assemblies based on each binding mode (see below). Post-processing allows retaining only the most favorable binding modes for regular self-assembly of the monomers. The generated structural families can then be investigated in terms of their local interface variability. To this aim, the interface is sampled at a finer scale using a combination of targeted docking and Monte Carlo exploration (described in the next sections).

### Sampling protein modes of interaction

Interaction modes were sampled via docking studies of the monomers, which produce solutions representing candidate pairwise interfaces ranked in terms of a scoring function or interaction energy [40, 51]. We used the PTools/ATTRACT suite for performing and analyzing docking simulations using the ATTRACT protocol [49, 50]. ATTRACT uses a multi-minimization strategy and a reduced (coarse grained) representation (about four heavy atoms per grain) for target proteins and/or nucleic acids. The force field governing the docking process is composed of a Coulombic term screened with a distance-dependent dielectric function and a smooth, 6–8 van der Waals term [50]. Several new functionalities were added to PTools/ATTRACT, coupling it to the Heligeom analysis described below (see also S2 Protocol, supporting information).

In this work, the monomers were exclusively considered and treated as rigid bodies, although limited flexible docking is possible with ATTRACT [49], and the approach described here is independent of the docking protocol. We performed two different types of docking simulations. In the first, one monomer (the *ligand*) was initially placed in a quasi-spherical array of regularly distributed starting points and orientations around the other (the *receptor*), at a constant distance between the two protein surfaces and with a 10 Å distance between neighboring points (S2 Protocol, supporting information). This allowed global identification of potential modes of association. Additionally, targeted docking simulations were performed in order to explore the variability of particular association geometries; for this the starting points were positioned in a dense grid (2 Å separation between neighboring points) no farther than 20 Å from the center of mass of the ligand in the particular geometry. This allowed detailed characterization of selected binding modes (see below).

A graphical view of the overall docking results was obtained by attributing to each residue of the receptor or ligand the best interaction energy of the interface or interfaces in which it was involved in the docking.

### Screw transformations with Heligeom

Heligeom is a Python module that interfaces with the Python/C++ library PTools [31, 49]. It is packaged with the latest version of PTools and includes a variety of scripts of varying complexity, including those developed for the present study (Boyer et al., manuscript in preparation).

The fundamental operation of Heligeom centers on the definition of the screw transformation. In general, the coordinates of a given monomeric unit can be derived from those of another through such a transformation [52], defined by the position O and direction Ω of a screw axis, a rotation of angle *θ* around this axis and a translation value *trans* parallel to the axis (shown schematically in S2 Fig, supporting information). Regular repetition of the screw transformation generically leads to a helical shape. Global parameters describing helix shape, *i.e*. the pitch (*P*), the number of monomers per turn (*N*) and the direction of rotation (*dir*), are derived from the screw parameters as follows:

$$\begin{array}{c} \begin{array}{ccc} N & = & \frac{360}{\theta }\\ P & = & N\times \mathrm{trans}\\ \mathrm{dir} & = & \left\{\begin{array}{c} R\;\mathrm{if}\;(\theta \times \mathrm{trans})>0\\ L\;\mathrm{if}\;(\theta \times \mathrm{trans})<0 \end{array}\right. \end{array} \end{array}$$

The pitch and the number of monomers per turn can be directly compared to values extracted from electron microscopy images where available. Analysis of a monomer-monomer pair with Heligeom typically consists of using a single command to automatically extract the screw parameters (helix axis, rotation angle and translation) from the coordinates of two interacting protein monomers, via an analytical geometric calculation [31, 52]. The structural data for the interaction may have been obtained experimentally or else through modeling or docking studies. In addition to extracting screw parameters, the same Heligeom command can generate fiber structures of arbitrary length.

Heligeom can also be used to simulate the assembly of monomers along a non-linear path. Once the screw transformation has been defined from the structures of two interacting monomers, oligomeric assemblies can be reconstructed along any given curved axis. Details can be found in S1 Protocol of the supporting information.

In the present study, scripts using Heligeom have been developed, either as post-processing tools to extract screw parameters and build fiber models from ATTRACT docking simulation output, or to directly incorporate screw analysis into sampling procedures such as Monte Carlo exploration (see below).

### Processing and filtering the sampling results

Automatic extraction of screw parameters from a PTools/ATTRACT docking output file (here typically about 50,000 poses) was readily performed with Heligeom. Additional filtering of the binding modes was performed to select modes of association compatible with the formation of helical assemblies. A first selection criterion (local filter) is the docking interaction energy. We retained modes of association up to 20 RT above the reference form interaction energy, which allows uncertainty due to the absence of the flexible regions of the monomer.

After initial screening, we applied a screen designed to identify binding modes suffering from non-local steric clashes arising from non-neighboring monomers due to the regular filament assembly. Binding geometries output by ATTRACT are characterized by Heligeom in terms of the number of monomers per turn (N) and pitch value (P) of associated regular assemblies, so these parameters were used for the classification. Structures with essentially no steric constraints are classified into the “Filament” category. To identify these geometries, the current version of our screen uses a simple radius-based scheme, allowing *P* > 2*R*_*M*_, where *R*_*M*_ is the maximum radius of a monomer. Similarly, a “Cyclic” geometry is defined if N falls in a range centered on an integral value ±0.1 and *P* is in the range 0 − 0.5 *Å*. Both “Filament” and “Cyclic” structures are passed through the screen with no further modification. On the other hand, “Near-cyclic” geometries are defined by accepting a pitch error of up to 5 Å per interface, and thus include structures for which *P* < (*N* − 1) × 5Å (S1 Fig, supporting information). These geometries are shunted to an automated Monte Carlo energy-minimization or “adjustment” procedure during which cyclic geometry is enforced (described in S1 Protocol, supporting information). The remaining monomer-monomer binding geometries, classified as “Near helical”, are currently tested for steric clash, as identified by the interaction energy computed between any monomer *i* and the two closest monomers from the next helical turn, *i* + *M* and *i* + *M* + 1, where M is the largest integer not greater than the number N of monomers per turn. Suitable adjustment methodologies to optimize such multiple interfaces will be applied in a future version of our approach.

### Exploring the variability of binding modes

In addition to identifying binding modes using unbiased docking, the variability of a given binding mode was explored as follows. First, targeted docking simulations were performed near the structure of the desired binding mode. Post-processing allowed associating screw parameters (pitch values, number of monomers per turn) with each resulting pose, together with the f_NAT_ with respect to the reference binding mode (see S2 Protocol, supporting information, for a definition of the quantities f_NAT_ and f_IR_ used in docking evaluation). Then, results with f_NAT_ > 50%, interface C*α*-RMSD < 3.5 Å and energy < −37 RT (native interface energy + 5 RT) were filtered out and used as starting geometries in the second stage of exploration. This second stage consisted of 10^5^ steps of Monte Carlo simulation at a temperature of 300K, using six variables (three translations and three rotations) for the rigid body displacement of the sampled (ligand) monomer with respect to its fixed (receptor) monomer partner. The Monte Carlo trajectory was confined to f_NAT_ values higher than 0.5 with respect to the reference geometry. Uniform sampling was performed in intervals of ± 5 degrees for the rotational variables and ± 3 Å for the translations. Acceptance rates varied between 0.2 and 0.4. Parameters such as helical pitch, number of monomers per turn, C*α*-RMSD, f_NAT_ and f_IR_ values with respect to the starting geometry were output at each simulation step.

### PDB files

Coordinate data was obtained from the Protein Data Bank [53]. Two crystal structures of RecA were used, with PDB codes 2REB [21] and 3CMW [23]. These structures are considered to be representative of the two known forms of the RecA filament: inactive or compressed (observed in the presence of ADP or without any cofactor) and active or extended (observed in the presence of non-hydrolyzable ATP analogs and with bound DNA) [14]. RecA in these two crystal structures presents large geometry differences at three locations: the N-terminal domain (residues 1–37) and the loops L1 (residues 156–165) and L2 (residues 194–210), which are disordered in the 2REB structure. These regions, together with terminal residues 1–5 and 329–333 which are disordered in 2REB, were pruned before rigid body docking was performed (next section). The intrinsically disordered C-terminal residues 324–352 of the RecA protein are not present in either the 2REB or the 3CMW crystal structures. The remaining monomer core structures, respectively referred to as 2REB_core_ and 3CMW_core_ differ by less than 1 Å C*α*-RMSD.

Structure 2REB was solved at a resolution of 2.3 Å in space group *P*6_1_ with no bound DNA or cofactor. It is very similar to the structure with PDB code 1REA, which was obtained with ADP as a cofactor but which contains only C*α* atoms; the C*α*-RMSD (root mean squared deviation) between the two entries is 0.3 Å. For this reason, the 2REB structure has been assimilated to the inactive form of the filament. The 3CMW structure consists of 5 monomers expressed as a single-chain fusion protein (space group *P*2_1_2_1_2) and was crystallized with ADP-AlF_4_-Mg, a non-hydrolyzable analog of ATP, in the presence of DNA with a resolution of 2.8 Å.

In this work, we will call X and X* the monomer-monomer binding modes in the crystal structure 2REB and 3CMW, respectively. Both forms correspond to right-handed helices, but they differ in pitch and monomer orientation with respect to the axis. Specifically, the pitch in structure 2REB is 85 Å for 6 monomers per turn, while it is 94 Å for 6.2 monomers per turn for 3CMW. We used the 2REB asymmetric unit and the crystal symmetry information provided in the PDB file to construct the X binding mode. In 3CMW, the five RecA fusion-protein monomer domains in the asymmetric unit are indexed by residue number: 1–333 for the first domains, 1001–1333 for the second, and so on. We separated these into monomers 1 to 5 for this work. Since their 3D structures differ slightly from one another, we used monomers 2 and 3 to define the X* interaction mode, with monomer 3 as the reference structure in order to reduce any end effects; in addition, the helical parameters that correspond to the dimeric association between monomers 2 and 3 match the helical parameters of the pentameric repetition. The filament form that was built from these two monomers can therefore be regarded as the regular form which is closest to the pentameric crystal structure. By extension, we will respectively refer to the filament forms resulting from RecA regular self-assembly with X or X* binding modes as the X and X*filament forms.

### Application of the PTools/Heligeom approach to RecA

**Fig 2:**
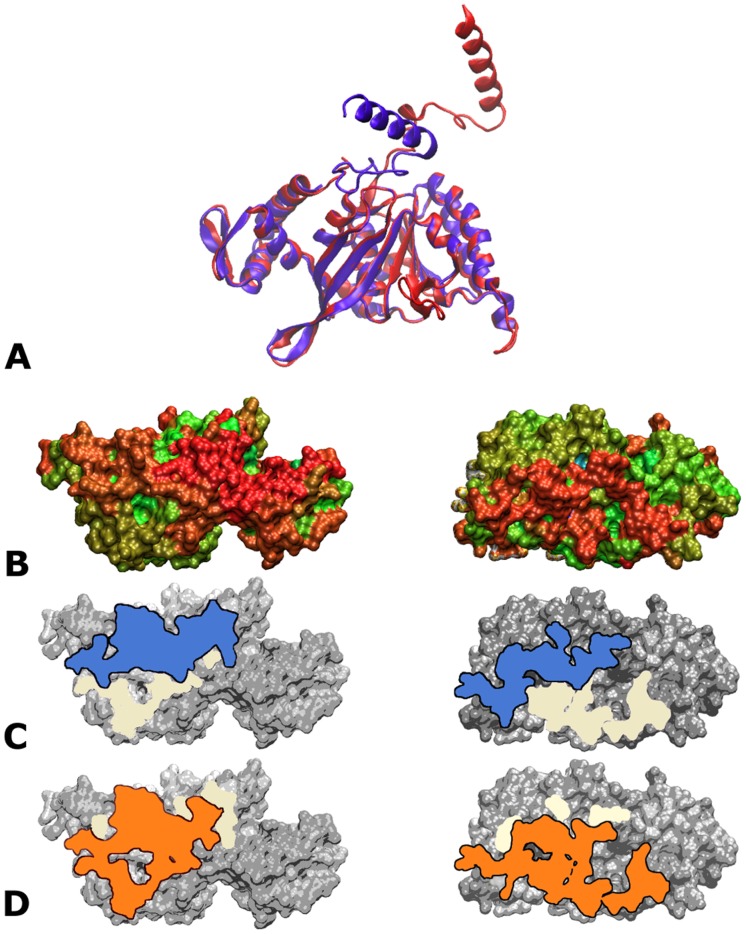
RecA-RecA docking. (A) Superposition of monomers taken from the 2REB (blue) and 3CMW (red) crystal structures, in cartoon representation; the rigid core is the region where the two structures are almost superimposable. (B-D) Binding regions on the surface of one RecA monomer, restricted to its rigid core. The left side shows the top of the monomer, the right side the bottom. (Note: orientations are chosen to best show the interacting surface, and are not exactly 180^∘^ apart). (B-C) Interface patterns characterizing the X (B) and the X* (C) binding modes. In both panels B and C, the union of the two interfaces is shown in pale yellow, while the specific X or X* interface is overlaid in blue (B) or orange (C). (D) Each residue is colored according to the best interaction energy of the interfaces to which it belongs, normalized by the best interaction value found in the simulation. The color ranges from blue to red to indicate 0–100% of the maximum interaction value. The interaction energies result from merged docking simulations carried out using the 2REB_core_ or the 3CMW_core_ monomers (see Methods). The rigid core represented as a 3D support in all views is 2REB_core_.

The approach was applied to the rigid cores of structures 2REB and 3CMW (2REB_core_ and 3CMW_core_). As detailed above, these structures are very similar and differ mostly by the conformations of surface side chains. Each of the structures was submitted to docking simulations and the results of both simulations were merged for analysis with Heligeom. We note that since the structures we used as starting point form specific binding geometries in the crystal complexes, our results could thus be construed globally as being “bound-bound”. However, the interface regions in these two assembly modes share no native contacts (see Table 2), and thus are largely “unbound” with respect to the other form. Indeed, no qualitative differences were observed in docking simulation results obtained using these structures (the correlation between the individual residue docking scores calculated as shown in Fig. 2 was 0.86).

Using 2REB_core_ structure we were able to generate binding modes close to X* (from 3CMW) (RMSD 4.8 Å, interface RMSD 4.2 Å, 50% common contact pairs), and vice versa (RMSD of 4.8 Å, interface RMSD 2.8 Å, 80% common contact pairs), with an energy difference of less than 4.2 RT from that of the native crystal binding mode in both cases.

Because of the restriction of our study to the central core of RecA, a supplementary filter was added to those described above in order to eliminate poses in which a residue bordering a flexible region (residues 38, 156, 165, 194, 210) were found in either of the two interfaces (i.e., with the preceding and following monomer).

Typical running times, for the RecA system, of the different stages of our approach can be found in S2 Protocol, supporting information.

### Calculating interface area contributions

The buried surface area (BSA) was calculated using atomic solvent accessible surface area (ASA) values computed by NACCESS [54] on both the separated monomer structures and their assembled forms, using a default solvent probe radius of 1.4 Å. The contributions of the different regions of the protein were obtained by repeating the BSA calculations before and after truncation of the concerned region and subtracting.

## Results

The integrative approach we take to the study of RecA assembly modes is illustrated in Fig. 1. Starting with the 3D structure of a single RecA monomer, we performed docking studies to identify favorable monomer-monomer binding modes, followed by the construction and analysis of corresponding helical assemblies with the new tool Heligeom. As two different crystal structures of RecA filaments (ADP and ATP forms) were employed in our work, we first defined a common “core” region (see Methods), that was then used for the rigid-body docking simulations. Thus, before presenting our detailed results on fiber morphologies, we first discuss the known RecA assembly modes in order to justify the use of the rigid core region, and to place our explorations in context.

### Characterizing known RecA assembly modes

Fig. 2A shows the 3D superposition of RecA monomers taken from the 2REB and 3CMW structures. The C*α* RMSD between the two structures is 6.7 Å, but this mainly reflects the movement of the N-terminal domain (residues 1–37, called here the Nter flexible region). On excluding these as well as of a number of smaller regions with undefined structure in 2REB, the RMSD falls to 0.89 Å for the “rigid cores” 2REB_core_ and 3CMW_core_ (see Methods).

These structural differences are accompanied by differences in the modes of RecA self-assembly, respectively noted X and X* (Methods). Indeed, when comparing two different dimers assembled according to X or X* after superimposing the first monomer, the C*α* RMSD of the second monomer is 22.6 Å. In comparing the accessible surface buried in the X (2REB) versus the X* (3CMW) interface (Table 1), it can be seen that the BSA includes contributions from both rigid and flexible regions of the interacting monomers. Table 1 also indicates that loops L1 and L2 contribute significantly to the interface area in the X* form (and almost equally, data not shown), while in the X form the fact that the loops are disordered suggests that they do not stably contribute to the interface. The C-terminal domain was not seen to contribute to the interaction in either the compressed or extended forms. The contribution of the Nter flexible region is large, on the order of the surface buried by the rigid core region. We point out that the total area buried in the RecA fiber is quite high—2800 to 4400 Å^2^. The BSA of the Nter flexible region alone, or of the core region alone, corresponds to the surface buried by one partner in a typical protein-protein interface (1600±400 Å^2^) [55]. Thus, the formation of native interactions involving either the flexible Nter or the rigid core region alone may suffice to stabilize the initial form of the complex.

**Table 1:** Contributions to buried surface area (BSA) in 2REB and 3CMW.

**Form**		**Total** ^a^	**Flexible** ^a^		**Rigid** ^a^	
			*Nter* ^b^	*Loops* ^b^	*Central core* ^b^	*f*_IR_ c
2REB	A	1400.3	709.0	–	691.3	0.71
	B	1377.8	664.5	–	713.3	0.61
3CMW	A	2225.5	895.1	387.1	943.1	0.59
	B	2171.9	840.3	398.6	933.1	0.44

^a^ Surface areas (Å^2^) of half-interfaces associated with each monomer A and B.

^b^ Region boundaries (detailed in Methods) were defined in order to minimize the RMSD difference between the two superposed rigid cores. Area differences with respect to reference [23] reflect differences in region definitions in addition to the neglect here of cofactor contributions.

^c^ *f*_IR_ reports the fraction of rigid core residues from monomer A or B belonging to the interface in both 2REB and 3CMW forms.

The similarity of two assembly modes can be quantified using the complementary measures f_NAT_ and f_IR_ (see S2 Protocol, supporting information). The f_NAT_ measures the fraction of contacting residue pairs (one residue from each monomer) that are shared between the two assembly modes. Considering the complete RecA protein, the f_NAT_ between the X and X* forms is 0.34; that is, 34% of the interface residue pairs are shared between the two modes. A complementary measure is f_IR_, defined by the fraction of interface residues (and not residue pairs) on a given monomer that are shared between the two modes, and which is thus less stringent than f_NAT_. The f_IR_ values of the interface overlap between the two modes is between 63% and 96% for the complete RecA monomers.

We next determined the relative contributions of the rigid core region and the Nter flexible region to the f_NAT_ and f_IR_ values. In Fig. 2, panel B shows the rigid core interface in the X form in blue and panel C that of the X* form in orange. The substantial overlap of the binding regions of the two forms seen in this figure corresponds to elevated f_IR_ values (44–71%) given in Table 1. However, no pairwise contacts between the rigid cores of adjoining monomers are conserved (f_NAT_ = 0). This is due to the different relative orientations of the neighboring monomers in the two forms.

In the crystal structures, the N-terminal helix (residues 6–23), because of its flexible attachment to the core, can be seen to maintain essentially the same interactions with the adjacent monomer in both the compressed and extended forms of the fiber [23, 28, 56]. Wang and collaborators [19] have observed similar characteristics in crystal structures of RecA homologs. More exactly, comparing the N-terminal helix interaction between RecA fiber forms, the calculated fraction of pairwise contacts (f_NAT_) is 0.9. We note that this region alone accounts for the overall total f_NAT_ value of 0.34. Because the relative orientation of the core region changes between the compressed and the extended forms of RecA, the long segment linking the N-terminal helix to the rigid core modifies its conformation in adapting to the geometry change.

Taken together, these observations suggest that the quaternary organization of RecA oligomers, to a first approximation, relies on the interface between the rigid cores of adjacent monomers. We therefore limited the investigations presented below to the rigid core of RecA.

### Investigating geometries of RecA autoassembly

We first carried out docking simulations to explore the diversity of RecA association modes using the ATTRACT method [31, 49, 50]. The docking was restricted to the rigid core of the RecA monomers, which as we noted above does not vary more than 1 Å between the different known helix morphologies. Two docking runs were carried out using the rigid cores obtained from the crystal structures 2REB or 3CMW, and the results merged for analysis (see Methods and S2 Protocol for more detail). Each pairwise interface geometry resulting from the docking simulation corresponds to a unique form of RecA oligomer assuming regular association. We examined these via Heligeom, which was employed to automatically characterize the geometry of the oligomers in terms of pitch, direction of rotation and number of monomers per turn, and to construct the corresponding fiber of arbitrary, specified length.

According to our working hypothesis, exploring the interface between rigid cores should permit recovering the known X and X* forms of association. We first checked that this was the case. The overall results of the docking are represented graphically in Fig. 2(D), in which each residue is colored as a function of the best interaction energy among all predicted interfaces involving it. The docking simulations predicted favorable interface regions that largely overlapped those characterizing the known interaction geometries (Fig. 2B and C), while sampling nearby alternatives as well. Indeed, the X and X* forms were accurately predicted by the simulations, with f_NAT_ = 94% and 89% native pairs of amino acids recovered respectively in each case. The corresponding C_*α*_-RMSD, calculated after superposition of the first monomer of the corresponding dimers, was 1.0 Å for the X form (2REB) and 2.4 Å for the X* form (3CMW). Moreover, these geometries were ranked among the best predictions of the docking simulations in terms of energy. These results validated our approach and led us to examine in more detail alternative association geometries predicted by the docking simulation.

Our analysis with Heligeom also allowed ascertaining if the association mode was consistent with regular helical or cyclic morphology, or if a steric clash was produced. While severely conflicted geometries were set aside, near-cyclic assemblies corresponding to the latter case were optimized using symmetry constraints (see Methods). S4 Fig (supporting information) shows plots of the raw results of this procedure for RecA. Each point in this plot represents the number of monomers per turn (N) and pitch (P) for a distinct monomer-monomer association geometry, Fthus forming an overall signature. The points along the horizontal axis correspond to optimized cyclic geometries.

**Fig 3:**
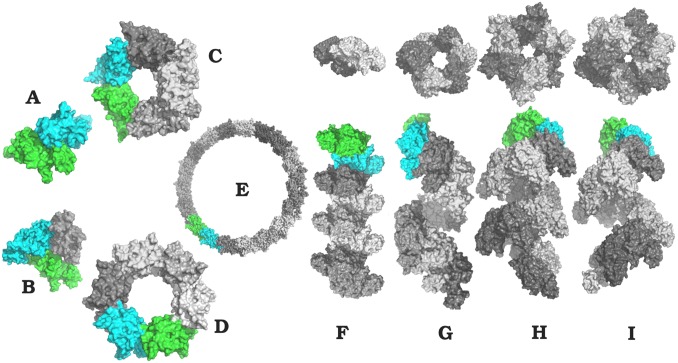
RecA self-assembly. Modes of regular association of RecA monomers resulting from docking simulations with ATTRACT. Structures labeled A to E represent cyclic assemblies, structure F is nearly straight, structure G is a left-handed helix and structures H, I are right-handed helices corresponding closely to the 2REB [21] and 3CMW [23] crystal structure forms (binding modes X and X*), respectively. Complete Heligeom characterizations of these structures are provided in Table 2.

A gallery of fiber organizations for RecA association is shown in Fig. 3, obtained from the simulations following S2 Protocol (supporting information). Complete Heligeom characterization of these binding modes, as well as of the X and X* binding modes, is provided in Table 2. The assemblies represented in Fig. 3 were chosen to illustrate how variations in the mode of RecA association with reasonable energies can result in drastically different geometric characteristics for the assembly, with all types of screw transformations, either cyclic (A-E), quasi-straight (F) or helical (G-I), being represented. Docking results characterized by a quasi-null pitch (A-D) gave way to dimers, trimers, pentamers or hexamers. An 18-mer quasi-ring structure (with an axial closure defect of only about 1.8 Å) was also obtained (structure E). Each of the forms A-I was verified to accommodate the pruned flexible regions, *i.e*. the two loops and the Nter flexible region, without steric clash (S2 Protocol and S5 Fig, supporting information). In addition, we verified that the monomer region involved in the N-terminal helix interface in both the 2REB and 3CMW crystal structures was accessible for binding.

**Table 2:** Comparison of RecA assembly modes.

**Mode** ^a^	**pitch** ^b^	**N**	**radius** ^b^		**E**_*int*_^c^	**f**_NAT_^d^	
			int	: ext		X	X^⋆^
A	0.0	2.0	0.0	: 46.6	−22.5^e^	0.0	0.0
B	0.0	3.1	0.0	: 53.6	−22.7	0.0	0.0
C	0.3	5.0	16.6	: 58.2	−33.9	0.0	0.0
D	0.1	6.0	20.7	: 62.9	−30.7	0.0	0.0
E	1.8	18.0	107.0	: 149.8	−30.2	0.0	0.0
F	52.5	2.0	0.0	: 46.3	−44.8	0.0	0.0
G	106.3	5.8	13.3	: 48.7	−40.2	0.0	0.0
H	72.8	5.8	10.9	: 61.6	−39.4	0.94	0.0
I	90.3	6.4	8.0	: 60.1	−41.1	0.0	0.89
X	82.7	6.0	11.7	: 62.9	−41.3	1.0	0.0
X*	94.2	6.2	6.2	: 58.9	−41.2	0.0	1.0

^a^ Each mode A-I describes one assembly type from ATTRACT docking experiments restricted to the rigid core of the RecA monomer. Last two lines provide corresponding information for the reference crystal forms X and X*; interaction energies *E*_int_ for these entries have been calculated between 2REB_core_ and 3CMW_core_ respectively, after energy optimization.

^b^ Value in Å

^c^ Per-interface energy (RT units)

^d^ measured with respect to the rigid cores in 2REB and 3CMW, respectively

^e^ the per-interface energy of the dimer (A) corresponds to half the value in the ATTRACT output (S2 Protocol, supporting information).

It can be emphasized that evidence for several of these predicted geometries has been observed experimentally—through atomic force microscopy [18] (forms A and D), and electronic microscopy [17, 20] (forms D, H and I), in addition to crystallography [21, 23] for forms H and I as described above. We also identified left-handed helices with very good interaction energy values (geometry labeled G), which can be related to left-handed forms of RadA observed by electron microscopy and crystallography [19]. Wang and coworkers [57] have suggested that left-handed assembly may be a general property for RecA family proteins. On the other hand, the quasi-straight geometry F, ranked second in terms of interaction energy, has not been observed in natural fibers, perhaps due to its lack of compaction.

We note that helices H and I rank among the best energy predictions, which in some sense conforms to their similarity to the known binding modes. However, the interaction energy alone here cannot be used to rank the most likely structures in terms of probability, which is a function of the change in free energy of the interaction. Also, as we have emphasized, only the rigid-core regions of the RecA monomers are used in these simulations. Further, as our interest is principally in filamentous assemblies, we focus on the interface energy only, while, for a given complex, the binding energy must also include contributions from all interfaces present in the assemblage. Relatedly, locally favorable monomer-monomer association geometries may be inconsistent with viable regular fiber geometries. For example, the lowest interface energy obtained from the pairwise monomer docking (structure Z, S2 Protocol of supporting information) would produce steric clashes between monomers of successive turns in a regular helix. Steric clashes in such cases could be resolved using multidocking techniques, just as we adjusted near-cyclic geometries to cyclic ones using energy minimization and symmetry contraints. However, even without adjustment, shorter stretches of fiber employing such interfaces could also play a role in mixed-mode fibers, as will be discussed below.

We compared the monomer interfaces for the different association modes in Fig. 3 in a pairwise manner. The f_NAT_ comparison showed that only the interfaces associated with ring morphologies C and E overlap somewhat, sharing 15% of their residue contacts, while those associated with the quasi-straight and left-handed helical filaments F and G share only about 3% of the residue contacts. All the other interfaces are perfectly distinct (0% shared contacts). The variability of the observed shapes presented in Fig. 3 therefore arises from the employment of distinct interfaces. However, it may also be the case that slight modifications in the interface can produce substantially different filament morphologies. An example of this is seen in the case of the RecA homolog Dmc1, in which a helical assembly mode shares 61% of interface contacts with the octameric ring assembly mode observed crystallographically, and with almost the same interface energy (S6 Fig and its caption, supporting information). This result is particularly interesting in the context of possible interconversion between such forms in Dmc1 and RadA [9, 10].

### Variability within selected families of binding modes

We next explored the variability of each of the two known binding modes, X and X*, by coupling Heligeom to targeted docking and Monte Carlo (MC) exploration, as explained in Methods. For each binding mode, we first performed targeted docking using a cloud of starting monomer ligand positions centered on the known interface. Poses that were obtained close to the known structure were then selected as starting points for subsequent MC simulations in order to map out possible solutions in the vicinity of the binding modes in detail (see Methods). Among the X and X* results, shown as blue crosses in S7 Fig (supporting information), 18 and 4 representative poses, respectively, were selected for this MC sampling.

**Fig 4:**
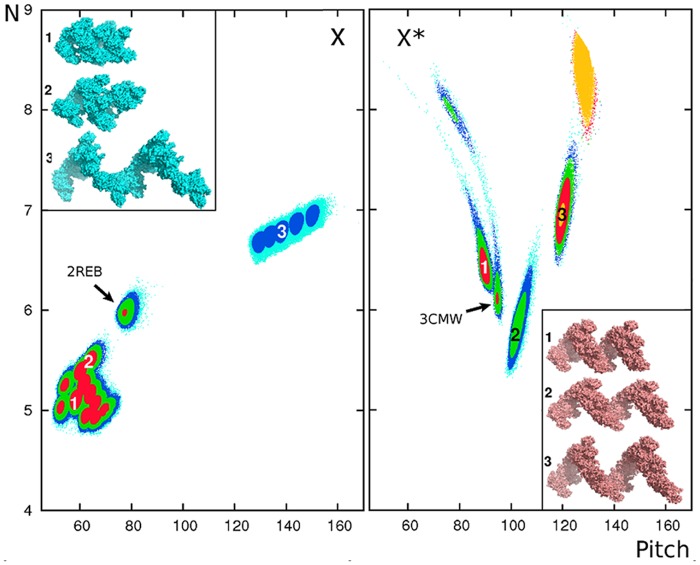
Exploration of the RecA-ADP (X) and RecA-ATP (X*) structural families. Helical characteristics of samples obtained via bound-bound docking simulations targeted to particular binding modes X (left) and the X* (right) followed by Monte Carlo exploration (see Methods). Sampled geometries are represented by the number of monomers per turn (N) versus pitch (in Å). The results are colored according to the interaction energy *E* in RT units, with *E* ≤ −42 (orange); −42 < *E* ≤ −40 (red); −40 < *E* ≤ −38 (green); −38 < *E* ≤ −36 (blue); *E* > −36 (cyan). MC sampling runs starting from the exact X or X* binding modes as extracted from the corresponding crystal structures are indicated by arrows. Inserts show representative sampled structures for each binding mode. As an indication of the degree of correspondence between the sampled geometry and the targeted binding mode, angular deviations (calculated using Heligeom) from the targeted binding geometry X or X* for the representative samples labeled 1, 2, 3 are 14.2, 5.2 and 23.3^∘^ (from X) and 5.1, 11.0 and 9.3^∘^ (from X*), respectively. As angular deviations are measured with respect to a local screw axis in each case, only their absolute values are globally meaningful.

Fig. 4 shows the sampled regions in terms of pitch and number of monomers per turn for the X (left) and X* (right) binding modes; for comparison, the figure also displays the results of MC simulations performed under the same conditions but starting from the exact binding modes (arrows in Fig. 4). The MC simulations show that each pose lies at the bottom of a stable energy well after the energy minimization performed as part of the docking procedure, and whose steepness is certainly exaggerated due to the rigid-body approximation. Broadly, however, each binding mode is seen to be associated with a family of low-energy right-handed helices whose members present similar interfaces to that of the targeted binding mode but with pitch values ranging from 45 to 160 Å for the X binding mode or from 70 to 140 Å for the X* mode. Representative members of the X and X* families are represented in Fig. 4 (inserts). The two binding mode families of X and X* occupy nearby regions of the N versus P plots, due to the projection of the multidimensional orientational configuration space onto the two dimensions N and P; in fact, as reported above, the two modes are completely distinct in terms of monomer-monomer contacts.

The results are compatible with EM observations obtained by the Egelman group through three-dimensional reconstruction specific to helical polymers [20, 58], in which the authors observed a large range of pitch values for helix families related to the compressed (ADP) or the extended (ATP) forms, with overlapping pitch values. Overall, we observe that the most stable elements of the X family present pitch values below 80 Å.

Finally, and unexpectedly, the modes of helix distortion revealed by Fig. 4 within the X or the X* families differ notably. For the X family, increase of pitch is accompanied by a roughly regular increase in the number of monomers per turn, indicating global unrolling/stretching of the fiber form (1 → 2 → 3 in Fig. 4, left panel). No such regularity is observed for the X* family: the right panel shows steeper variations, with a slope that can be positive (pitch values above 100 Å) or negative (below 100 Å). This indicates that stretching (2 → 1 in Fig. 4, right panel) as well as compression (2 → 3) of X* fiber forms with 100 Å pitch are accompanied by an increase of the number of monomers per turn– that is, helix unwinding.

### Binding mode variations in a single fiber

**Fig 5:**
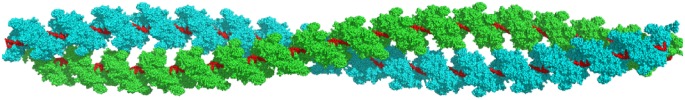
View of one turn of a RecA negatively supercoiled filament at atomic resolution. The structure has been constructed following the geometric characteristics described by Shi and collaborators [18], with a pitch *P* = 160 nm for the the supercoil (see text and S1 Protocol, supporting information). The axis of the two strands (in cyan and green, respectively) are separated by 110 Å. The DNA incorporated in each of the two RecA filaments is in red.

Variations in the binding mode within a single fiber can also lead to a variety of changes in filament morphology. For example, in the negatively supercoiled filament shown in Fig. 5, consecutive interfaces differ slightly from each other, allowing the torsional deformation to be regularly distributed along the whole structure. The pictured filament was constructed using PTools/Heligeom (S1 Protocol, supporting information), following observations obtained by atomic force microscopy [18].

**Fig 6:**
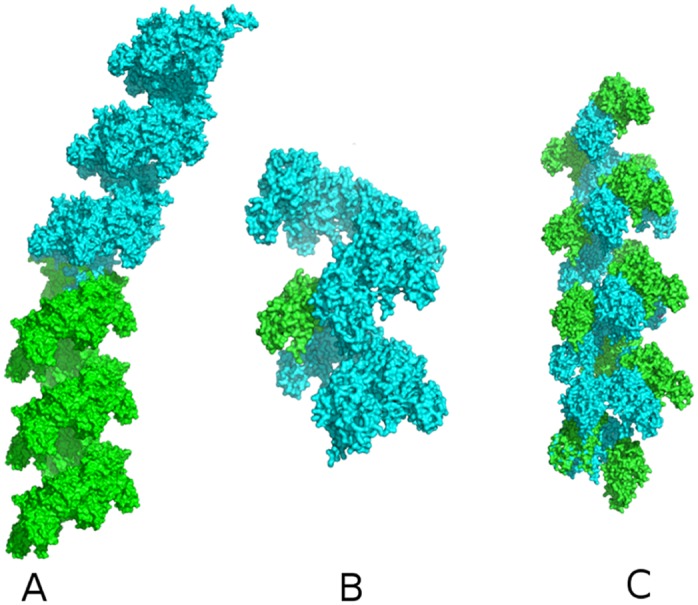
Models of RecA filaments with alternating binding modes. Consecutive monomers are represented in surface representation with different colors. (A) The model features a junction between X*-form regions (monomers 1–18) and X-form regions (18–36). (B) The filament, principally in the X* form, presents X-type interfaces periodically distributed every 6 monomers. (C) 24-mer filament with alternating X* and X interfaces. The monomers with an X-type upper interface are represented in green, those with an X*-type upper interface in cyan. Pitch values characterizing the helices in A and C and the superhelix in B are indicated.

Relatedly, it may be envisioned that binding modes belonging to different structural families coexist within a single RecA fiber. Alternation between fiber regions presenting extended or compressed forms has been observed by electron microscopy [56]. Fig. 6A shows a model of a X– X* junction, obtained with PTools/Heligeom by simply appending monomers using the different binding modes. Each junction results in a ∼35^∘^ kink, corresponding to the ∼35^∘^ difference between monomer orientations with respect to the helix axis in structures 2REB and 3CMW. We note that kinks can indeed be observed in Fig. 1 of reference [56].

In Fig. 6B-C, we explore the effects of different combinations of X and X*binding modes on the overall RecA fiber morphology. Such a chimeric structure is shown in Fig. 6B, in which an X interface is periodically inserted every six monomers in a filament otherwise in X* form. This corresponds to a situation in which ATP molecules would be hydrolyzed every six monomers (which appears to be the case in active RecA filaments [59, 60]), with the monomer-monomer binding modes modified accordingly. The result is a negative superhelix with a pitch of 312 Å, an external radius of 182 Å and 59 monomers per turn. On the other hand, a regular fiber is obtained when X and X*binding modes alternate evenly along the filament (Fig. 6C). In this case, the resulting geometry is intermediate between X and X* helices (82 Å pitch and 6.4 monomers per turn). The main characteristic of this structure is a locally reduced and strongly variable groove width with respect to the X and X* forms (see Figs S8, S9 and S3 Protocol, supporting information). We note from S9 Fig that the X* form itself presents large and regular groove accessibility, which could be related to its co-protease function during the SOS response [60–62]. Vanloock and collaborators have shown that upon binding the RecA filament groove, the LexA protein responsible for this function favors the “active” (X*) filament form, while protein RecX that inhibits recombination and also binds in the filament groove stabilizes the “inactive” form fo the filament [58]. Finally, although no direct proof of the existence of filaments with alternating X and X* interfaces has been published for RecA, such filaments represent putative transient intermediates during interconversion between ATP and ADP-forms of RecA filaments.

## Discussion

In this study, we have taken an integrated approach to investigating RecA supramolecular assemblies, by sampling possible modes of association via docking and Monte Carlo simulations coupled with helical analysis using a new software tool, Heligeom, in the PTools modelling package. The first result is that interactions between rigid core regions of successive RecA monomers suffice to account for all known oligomeric forms of RecA assembly, including those that have been postulated but never solved at atomic resolution such as left-handed helices.

An important role for the RecA rigid core in filament morphology was expected because of its large contribution to the interface area in the 2REB and 3CMW forms (Table 1). However, because flexible regions contribute by approximately the same amount, the central role of the rigid core needed to be established. The building-block role for the RecA rigid core region gives latitude to flexible and mobile regions of the protein [63], single side-chain mutations [64] as well as external factors (ATP, ions, DNA [65] or associated proteins [58]), to modulate energetic preferences among binding modes and possibly to actively control the passage between binding modes, as postulated by Chen and collaborators [19]. Our results suggest for example that the ATP cofactor stabilizes the X* form of RecA association (S2 Dataset and S7 Fig, supporting information). Karlin and Broccieri [66] proposed that the stabilization of the filament active form may involve the specific formation of inter-monomer salt bridges. Indeed salt bridges involving highly conserved residues Glu96-Lys250, Glu154-Arg176 (S1 Dataset and S3 Fig, supporting information) can be found deeply buried in the monomer-monomer interface in structure 3CMW, but not precisely in the way that was anticipated. More extensive calculations, using all-atom representations and explicitly taking into account solvation, will be necessary to determine the relative contributions of the different factors driving the filament towards one or another form. The present work is intended to contribute new elements for understanding the roles of such factors in future studies.

Our results also provide new understanding of the relationship between modes of RecA self-association. For example, we identified families of structures that could be considered close in terms of binding mode since they share interacting pairs of amino-acids (structures C and E in Fig. 3). The combined use of Monte Carlo sampling with Heligeom allowed us to explore the detailed variability of RecA filament morphology within such structural families, each defined as an ensemble of binding modes with low interaction energy and a fraction of common contact pairs (f_NAT_) greater than 50%. We caution that our approach does not permit ordering the structural families in terms of their probability of formation, which would require evaluation of the free energy of interaction, and would thus necessitate taking into account not only the flexible regions but also the system composition (ion, cofactor and monomer concentrations [65]).

Variability in the helical pitch of RecA filaments may also play an important role in binding DNA. During the process of homologous recombination, RecA filaments are found bound to DNA that is extended (by 50%) and unwound (by 40%). This corresponds to a helix with 18 base pairs per turn and about 94 Å pitch. The recent work of Bosaeus and collaborators [67] establishes that the DNA form in the RecA filaments corresponds to a metastable state of double-stranded DNA that is stabilized by its interaction with the recombinase filament. Small modifications of DNA stretching may then direct the DNA into either a B-DNA state or a S-DNA state. The DNA characteristics may also be modified during base pairing exchange. Adaptability of the protein filament geometry within a given family, similar to that seen in our targeted docking results, may allow the conservation of key interactions with the DNA during such events.

More generally, variability within a given binding mode enables an oligomer to stably undergo reasonable distorsion in response to physical influences from the environment. For example, in the 3CMW crystal structure, the binding modes locally defined by the five monomers (four interfaces) differ somewhat, with associated pitch values respectively comprised between 89.2 and 98.8 Å (Heligeom analysis; the average value is 94.7 Å). Similarly, the RecA helix in the crystal structure 1U94 is characterized by a pitch of 74 Å for 6 monomers per turn, even though its binding mode shares 100% of the interchain residue contacts with 2REB (pitch of 85 Å, also 6 monomers per turn). Finally, in the crystal structure of the RecA human homolog Rad51 determined by Conway and collaborators [68], two slightly different binding modes were reported to alternate along the helix. In any biological setting, RecA filaments are also not expected to conserve perfect symmetry. Thermal effects or dense packing in the cell [69] will introduce some degree of disorder. The persistence length of RecA filaments has been estimated to be about 900 nm when bound to DNA [70] and 95 nm when DNA-free [71]. In many cases, distortion induced by external constraints can be smoothly distributed over the whole structure, taking advantage of the local variations within the binding modes. An illustration is the response to torsional stress transmitted by bound DNA, as observed by Shi *et al*. [18] and as we modeled in Fig. 5. When the external stress is very large, or if regulating elements (cofactors, conformations of flexible regions) are not uniformly distributed along the fiber [60], different binding modes can be expected to coexist within single RecA oligomers. Examples of such multi-modal associations have been documented in the case of ring assemblies [72], where they have been attributed to the effect of non-uniform cofactor hydrolysis [73]. As shown in Fig. 6 for the case of RecA, a large range of variation in terms of filament morphology can result simply from multi-modal association. In this respect we note that kinks, strong curvature and other irregularities can be observed in EM images (see for example Fig. 3B in [74], Fig. 1 in [56] or Fig. 1 in [20]).

An advantage of using PTools/ATTRACT in this work is the possibility of easily incorporating coarse grained representations. In addition to sparing computer time, coarse-graining offers a simple way of smoothing the potential energy surface and implicitly accounting for small conformational changes at the interface. In earlier work it has been shown that the ATTRACT docking performance largely tolerates conformational changes of small and medium-sized side chains [50]. Side chain rotamer changes are particularly frequent upon formation of protein-protein interfaces [75]. Although not used here, ATTRACT’s handling of side chain or even loop flexibility at the coarse-grained level allows selecting side-chain rotamers or loop substates during the docking process [50, 76]. If the conformations of long interfacial side-chains need to be optimized at the atomic level, one may resort to the methods recently developed by the Baker [77, 78] or Redon [79] groups, which simultaneously optimize side-chain and even main-chain geometries within protein oligomers, together with the relative positioning of monomers. The two methods are specific to symmetric assemblies and take advantage of the symmetry to reduce the number of degrees of freedom. When symmetry is disrupted (for example due to axis curvature), one can use more general methods for optimization of interface packing as described for example in references [80, 81]. With PTools, elements that have been locally optimized can then be re-injected into the supra-assembly using simple superposition commands. Coarse grained representations are also compatible with the exploration of internal deformation of the monomers or concerted deformation of dimers, for example by following vibrational modes [82, 83]. This aspect has not been directly considered in the present study but can be easily coupled to docking or Monte Carlo explorations in order to evaluate such effects on the global helical form. Such internal deformations may contribute significantly to pitch variations. Methods for taking into account flexibility may prove important in studying recombinases such as RadA or Dmc1 from higher organisms because of the proportionally larger contribution of their flexible N-terminal domains compared to RecA.

Finally, our focus on interfaces makes it possible to construct non symmetrical morphologies and notably those combining different binding modes. This can be important given that biological processes often involve symmetry disruption. In this vein, we are currently investigating the morphology of RecA filaments presenting both ADP- and ATP-like interfaces in more detail. Our results obtained for RecA justify the coupling of docking simulations and Heligeom processing in interpreting EM or AFM observations of regular or irregular oligomeric assemblies. In its present state, our approach is limited to studying individual filamentous assemblies, i.e., in which there are predominantly *i*, *i* + 1 monomer interactions. Further addition of techniques from the multimolecular docking [31, 34] should enable its future application to study the morphology of assemblies of protofilaments such as cytoskeleton fibers and taking account interactions with auxiliary proteins.

## Supporting Information

S1 Protocol Filament construction with PTools/Heligeom. (PDF)

S2 Protocol Exploring RecA interfaces with PTools/Heligeom. (PDF)

S3 Protocol Measuring protein filament groove width. (PDF)

S1 Dataset Interface residues in the X and X* modes of association. (PDF)

S2 Dataset Influence of RecA flexible/mobile components on its binding modes. (PDF)

S1 Fig Scheme of geometric filtering. After energy filtering, binding geometries issued from ATTRACT docking simulations are characterized by Heligeom in terms of the number of monomers per turn (N) and pitch value (P) of associated regular assemblies, and are separated into “Filament” (blue), “Cyclic” (green), “Near-cyclic” (orange) or “Near-helical” (red) categories based on their corresponding position in the plot of *P* versus *N* schematized here. In the present work, the horizontal dotted line separating the “Filament” from the other categories has been set at *P* = 2*R*_*M*_, where *R*_*M*_ is the maximum radius of the monomer. The green boxed areas correspond to “Cyclic” geometries centered on integral values of *N* ± 0.1 with allowed values of *P* ≤ 0.5 Å. “Near-cyclic” geometries are defined by accepting a pitch error of up to 5 Å per interface, and thus *P* < (*N* − 1) × 5 Å. These geometries are shunted to an automated Monte Carlo energy-minimization (adjustment) procedure in which cyclic geometry is enforced.
(EPS)

S2 Fig Scheme of the Heligeom screw motion model between two monomers A and B. The screw axis is defined by point O and direction Δ. The transformation from monomer A to monomer B is the combination of a rotation *θ* around the axis and a translation *trans* along the axis.
(EPS)

S3 Fig Conserved surface residues. (Top) residues on the 2REB_core_ surface have been colored according to the sequence conservation index (CI) of 63 RecA proteins (Karlin and Brocchieri, J Bacteriology 1996, 178, 1881–1894); selected values are reported in Table S1–1 of S1 Dataset, supporting information; (bottom) the residues have been colored according to the docking results; reproduced from Fig. 2D, main manuscript.
(EPS)

S4 Fig Helical parameters from Heligeom for regular assemblies obtained from ATTRACT docking simulations in RecA. Each circle on the P versus N plot, in which P is the pitch (Å) and N the number of monomers per turn, represents one docking pose geometry after filtering and post-processing as described in Methods and Fig. 1 (main article), S1 Protocol and S1 Fig (supporting information). Pitch values for left-handed helical geometries (parameter *dir* in Methods) are given as negative values. Points are colored by the interface energy of the corresponding association geometry, with darker values indicating more favorable energies.
(EPS)

S5 Fig Accommodation of flexible regions in predicted RecA fiber forms. The fiber forms A to I (Fig. 3, main manuscript and SI S2 Protocol) are displayed in ribbon representation, with monomers alternatively colored in white or gray. The extremities of the flexible regions are shown in van der Waals representation, with the extremities of loop L1 (residues 156 and 165) in blue, those of loop L2 (194, 210) in red and the linker extremity (residue 38) in green. The N-terminal helix binding region (residues 89, 124, 127–128, 131–132, 135–138) is in orange.
(EPS)

S6 Fig Overlapping helical and cyclic forms of Dmc1. Comparison between the cyclic octameric form of Dmc1 (1V5W in green, Kinebuchi et al., Mol Cell 14, 363–374, 2003), and a helical form obtained using ATTRACT/Heligeom (violet) following the same procedure that was used for RecA. The construction and the present representation were restricted to the monomer rigid core (residues 99–270, 290–340) of 1V5W. Cartoon representations show the “receptor” and “ligand” monomers, both in yellow for the cyclic octamer and in yellow and violet respectively for the helical form. The two forms are superimposed using the receptor monomer. The two assembly modes share 61% of monomer-monomer contact pairs. Their interaction energies are within 1 RT, and C_*α*_-RMSD calculated for their ligand monomers is 5.5 Å. The number of monomers per turn N = 7.4 and pitch P = 79.0 Å characterizing the right-handed helical form are compatible with geometries seen in filaments active in homologous recombination.
(EPS)

S7 Fig Variability of the X and X* fiber forms in the presence of flexible/mobile interaction component. (A) Comparison of X (left) and X* (right) modes of monomer-monomer association. Two consecutive monomers issued from the PDB files 2REB (left) and 3CMW (right) are represented. In both cases, the top monomer is represented in surface mode, in white. The bottom monomer is shown in a ribbon representation and with an orientation that is common to both panels left and right. The rigid core is in grey; the L1 loop (right) or its extremities (left) are in blue; the L2 loop (right) or its extremities (left) are in red; the N-terminal domain, including a terminal helix and a flexible linker, are in green (right) or green and orange (left), the orange region corresponding to the fraction of the linker which folds back on its own monomer in structure 2REB (residues 30–37). The ATP cofactor in the right panel is in purple. (B) Results from targeted docking simulations on X (left) and X* (right) modes of association, characterized by their pitch P and number of monomers per turn N; the results were obtained in the presence (red +) or in the absence (blue ×) of flexible or mobile elements, the 30–37 linker segment for X (left) and the ATP cofactor for X* (right) (see S2 Dataset).
(EPS)

S8 Fig Groove width measurement. (A) Construction of a set of reference points used to measure the groove width of a protein filament. *r* and *R* are respectively the inner and outer radii of the filament, reference points are taken every 1 Å between (*r* + *R*)/2 and *R*. (B) Visual representation of the reference points (in red) used to compute the groove width of a RecA filament. Values are calculated every half degree. Further details are provided in S3 Protocol, supporting information.
(EPS)

S9 Fig Variation of the groove width along one filament helical turn. Groove width variations along one filament turn (360^∘^) are represented for the X filament form (black line), the X* filament form (red line) and the model with alternate X and X* interfaces (green line) (see S3 Protocol and S8 Fig, supporting information).
(EPS)
