# Supplementary material for: An Integrative Approach to the Study of Filamentous Oligomeric Assemblies, with Application to RecA
Source: PLoS One. 2015 Mar 18;10(3):e0116414. doi: 10.1371/journal.pone.0116414 (PMC4364692; doi:10.1371/journal.pone.0116414)
Supplement: S1 Protocol — (PDF) [file pone.0116414.s001.pdf]

Protocol S1: Filament construction with PTools/Heligeom

Screw movement

The screw transformation, which is the basis of the Heligeom analysis and construction capabilities, is defined by an axis (Δ), a rotation angle θ around this axis and a translation *trans* along this axis (Scheme S1-1) [1,2]. It enables relating a mode of association at the dimer level to the geometry of a corresponding multimer in which this mode would be regularly repeated. Helical parameters such as the pitch (P) or the number of monomers per turn (N) are readily obtained from the values of θ and *trans* as shown in Methods. Details of Heligeom script commands and further examples of their utilization are found in supporting information Protocol S2 and reference [3].

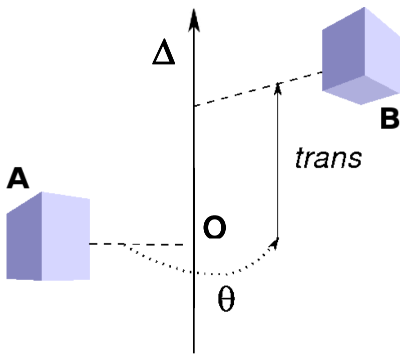

Scheme S1-1 - Definition of a screw transformation

Optimizing ring geometries

We consider that binding geometries resulting from docking simulations correspond to a cyclic organization when the number of monomers per turn *N* differs by less than 0.1 from an integral value and when the pitch increment per interface *P/*(*N*-1) is less than 0.5 Å. When *N* and *P* do not meet these conditions but the pitch increment is less than 5 Å, we presume that the helix geometry resulting from regular monomer association following this binding mode is sufficiently close to a ring geometry that the latter can be achieved with only minor adjustment ("Near-cyclic" organization in Figures 1 and S1, supporting information).

In such cases two ring geometries are attempted, comprising either *M* or *M*+1 monomers, where *M* is an integer such that $M \leq N < M+1$. The adjustment is readily performed within the formalism associated with screw transformations (see above). Given the proximity of the target ring structure to the initial screw transformation, we conserve the screw axis Δ. C_0 and C_1 refer to the centers of mass of the receptor and ligand defined in the docking simulation. R_i is the initial distance between C_0 and the screw axis, and θ_i the initial value of the rotation to pass from receptor to ligand.

The cyclic geometry is obtained simply by setting the *trans* value to 0 and the target angle θ_T to $2\pi/M$ for the M-member ring or $2\pi/(M+1)$ for the M+1-member ring. The distance R_i is accordingly modified to value R_T in such a way that the (C_0, C_1) distance is conserved between the initial and target geometries (see Scheme S1-2).

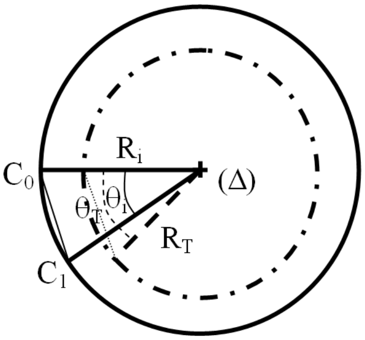

Scheme S1-2 : Adjusting the distance between the monomer and the axis

$$R_T \sin(\theta_T/2) = R_i \sin(\theta_i/2)$$

or

$$R_T = R_i \sin(\theta_i/2) / \sin(\theta_T/2).$$

Starting from the new distance R_T, we adjust the position and orientation of the receptor with respect to the fixed axis by performing a series of 1000 steps of Monte Carlo simulation where the receptor is displaced along the radial axis and rotated around three othogonal axes centered on C_0. Trial radial displacements are taken uniformly in the range [-3 Å, 3 Å] and angular variations in the range [-5°, 5°] in each of the three directions.

For the 90489 RecA docking poses, 640 binding geometries were considered as "Near-cyclic" and their adjustment to cyclic geometries gave rise to 1280 cyclic geometries with an average deviation of 10Å Cα-RMSD from the starting geometry. Among these cyclic geometries, 437 successfully passed the filtering process based on steric and energetic conditions (Methods, main manuscript). These geometries varied from homodimers (1 occurrence) to 38-mers (1 occurrence), with the highest populated cyclic N-mers found for N between 3 and 11 monomers per turn (> 10 occurrence). The number of occurrences for a given N value reaches 88 (N=4), corresponding to 56 families of distinct binding geometries (Table S1-1).

N	2	3	4	5	6	7	8	9	10	11	12
#adjusted	1	77	88	72	51	36	31	27	13	11	6
# families	1	52	56	47	42	31	26	22	11	10	6

Table S1-1: Distribution of the cyclic geometries resulting from the adjustment of RecA docking poses in terms of number of monomers N. *#adjusted* represents the number of cyclic geometries obtained from the adjustment+filtering process; *#families* is the corresponding number of structural families (within a family, pairs of members are characterized by fNAT greater then 50%, see main manuscript).

Construction around a curved axis

Helical oligomers can be constructed around any axis provided by the user, which can be input to Heligeom as a series of atoms positioned approximately 1 Å from each other. Generation of the filament is performed by successively applying the given screw transformation with respect to the axis at each monomer level.

Below we report the PTools commands used to construct the supercoiled RecA-DNA filament represented in Fig. 5 of the main article. The construction involves two intertwined regular helical assemblies of RecA proteins, each formed along a separate curved axis forming a negatively supercoiled turn. Such structures have been observed by electron microscopy and described in reference [4].

The following Python code generates the two superhelical axis portions (here stored as `axis1.pdb`, `axis2.pdb`), differing in the direction of the extension: one in the +*Z* direction and the other in the −*Z* direction. Their pitch, 160 nm, represents the lower limit of the pitch distribution observed in reference [4].

To generate the first axis portion, a point `r1`, initially situated 54 Å from the origin in the *X* direction (the approximate radius of the RecA filament in the PDB entry 3CMW), is incrementally displaced along the superhelical path in 1600 steps. The desired rotation at each step is produced using the PTools function `ABrotate()`, where points `A` and `B` define the *Z* axis. The generated points are stored in the `Rigidbody()` object `R1`. The second axis portion is generated from the first by rotating by π about the *Z* axis and flipping the result.

```
from ptools import *
import math

# generating first axis portion

r1 = Rigidbody()
r1.AddAtom(Atom(Atomproperty(),Coord3D(54,0,0)))
R1 = r1
A = Coord3D(0,0,0)
B = Coord3D(0,0,1)

dphi = 2.0*math.pi/1600
for i in xrange(0,1600):
    r1.ABrotate(A, B, dphi)
    r1.Translate(B)
    R1 = R1 + r1
WritePDB(R1, "axis1.pdb")

# generating second axis portion

R1.ABrotate(A, B, math.pi)
R2=Rigidbody()

for i in range(1600,1,-1):
    ato = R1.CopyAtom(i)
    R2.AddAtom(ato)

WritePDB(R2, "axis2.pdb")
```

A RecA helix can then be built along each of these two axes using Heligeom's `buildProteinAlongAnAxis.py` script.

```
python buildProteinAlongAxis.py axis1.pdb reca.pdb reca-n1.pdb >
                                                      RecAsupercoil1.pdb

python buildProteinAlongAxis.py axis2.pdb reca.pdb reca-n1.pdb >
                                                      RecAsupercoil2.pdb
```

This tool first obtains the local screw transformation parameters from the two structures provided (`reca.pdb` and `reca-n1.pdb`, successive monomers in 3CMW [5]). The filament is obtained by sequentially generating monomer `n` from monomer `n-1`, using a composition of the screw transformation with the transformations between appropriate sections of `axis1.pdb`. Construction can be accompanied by simultaneous rotation of the oligomer around the curved axis. This is necessary in general as the filament is no longer symmetric.

References

[1] Bottema O, Roth B (1979) Theoretical Kinematics. North-Holland Series in Applied Mathematics and Mechanics 24. North-Holland Publishing Company.

[2] Angelidis A (2004) Hexanions: 6D space for twists. In Tech rep Oxford University Computing Services.

[3] Boyer B, Basdevant N, Meng A, Poulain P, Robert CH, Ha-Duong T, Saladin A, and Prévost C (2014) Ptools : a multiscale modelling tools for biomolecular assemblies. In preparation.

[4] Shi W-X and Larson RG (2005) Atomic force microscopic study of aggregation of RecA-DNA nucleoprotein filaments into left-handed supercoiled bundles. Nano Lett 5, 2476-2481.

[5] Chen Z, Yang H and Pavletich N P (2008) Mechanism of homologous recombination from the RecA-ssDNA/dsDNA structures. Nature 453, 489-484.
