# Supplementary material for: An Integrative Approach to the Study of Filamentous Oligomeric Assemblies, with Application to RecA
Source: PLoS One. 2015 Mar 18;10(3):e0116414. doi: 10.1371/journal.pone.0116414 (PMC4364692; doi:10.1371/journal.pone.0116414)
Supplement: S2 Protocol — (PDF) [file pone.0116414.s002.pdf]

Protocol S2 : Exploring RecA interfaces with PTools/Heligeom

This section details the PTools/PyAttract and PTools/Heligeom commands that were used for the generation of various modes of auto-association of the RecA monomer, the analysis of each of these modes in terms of the corresponding screw transformations, and the construction of corresponding regular assemblies, either helix, ring or straight oligomer. PyAttract and Heligeom are two modules of the PTools library dedicated respectively to macromolecular docking and to analysis/construction of higher-order assemblies starting from the modes of association obtained through the docking.

Docking procedure

Preparation of a PTools/ATTRACT coarse-grained docking run necessitates the creation of simplified ("reduced") structures for the docking partners using the `reduce.py` script. Here, the 2021 heavy atoms of the rigid core of the RecA protein are reduced to 595 grains. In the following description, one partner (arbitrarily chosen) will be called the receptor and the other the ligand; the receptor is held fixed in the docking run. A total of 244 starting points were distributed around the (fixed) receptor using the `translate.py` script. From each starting point and for each of 228 predefined ligand orientations (rotations), the `Attract.py` script ran a series of six minimizations of the interaction energy between the receptor and the ligand.

In the present example, two docking simulations were run starting from the rigid cores of two RecA protein structures 2REB [1] and 3CMW [2] (see Methods). Four reference structures were given for comparison, obtained from the two different modes of filamentous association seen in the PDB entries 2REB and 3CMW. Each filament structure furnished two ligand orientations, corresponding to the `n+1` (upper) and `n-1` (lower) interfaces with respect to the receptor. For each of the two PDB entries, then, two reference files were obtained as follows. First, three consecutive (and non-terminal) monomers were extracted from the filament structure and superposed on the central monomer that was used as receptor in the docking simulation, using the PTools selection and superposition utilities. This defined the preceding and following monomer positions relative to the receptor, onto which the ligand molecule in the docking simulation was superimposed in order to create the corresponding reference structures. For each ATTRACT output, root mean square deviation (RMSD) values with respect to each of the four reference structures, taken on the C_α atoms, were calculated.

The rigid cores, $2REB_{core}$ and $3CMW_{core}$, were obtained by pruning the flexible regions (N-terminal domain, L1 and L2 loops, see Methods). They were reduced (`reca-rigid.pdb`) to coarse grained resolution (`reca.red`) using the PTools `reduce.py` script.

```
python reduce.py --prot reca-rigid.pdb  > reca.red
```

Docking was performed using the ATTRACT function, which performs energy minimizations between two partner macromolecules with respect to translation and rotation degrees of freedom, starting from thousands of initial configurations, in which the ligand is distributed around the receptor at different positions and orientations.

```
python attract.py reca.red reca.red --ref=reca_n1.red
     --ref=reca_n2.red --ref=reca_p1.red --ref=reca_p2.red > docking.att
```

In this command, note that the two molecules to be docked are identical (`reca.red`), and four separate reference structures have been provided for root mean square deviation (RMSD) calculations (calculated using C_α coordinates): two of them with the next monomer

(`reca_n1.red` for structure $2REB_{core}$, `reca_n2.red` for structure $3CMW_{core}$), and two with the preceding one (`reca_p1.red` and `reca_p2.red`).

Selected and annotated output lines from the ATTRACT simulation are shown here (annotations are indicated by "#").

```
#     i     j     E      rmsd1  rmsd2  rmsd3  rmsd4
==    9   186  -46.48   25.66  34.04  64.49  54.34 # Z
==   42   113  -45.00   55.09  49.00  73.41  72.06 # A
==   86    95  -44.78   63.68  68.37  37.08  35.44 # F
==   39    21  -41.14   28.03   2.43  70.45  67.62 # I
==   77    85  -40.21   52.23  59.15  31.22  19.62 # G
==   20   204  -39.41    0.96  26.52  71.06  59.94 # H
==   99   117  -33.91   79.33  73.69  52.21  58.13 # C
==   49   129  -30.69   65.99  54.31  70.20  72.65 # D
== 215   131  -30.16   73.53  75.56  58.45  54.67 # E
== 241    17  -22.68   79.85  65.17  60.28  71.69 # B
```

Each line represents an interface geometry after energy minimization carried out by ATTRACT, and contains, from left to right, two indices for the starting orientation in terms of translation and rotation, the value of the interaction energy (in RT units) and the RMSD values (in Å) corresponding to the four reference structures. On the right-hand side we have added structural labels that correspond to the labels discussed in the main article and displayed in Figure 2 of the main article and Figure S5 of the Supporting information.

Comparison of two binding modes

Two residues, one from each monomer, are considered to be in contact if any of their pseudo-atoms are within 7 Å [3]. Comparison of two modes of interaction was characterized by f_{NAT}, which is the fraction of residue-residue interface contacts in the first mode that are also present in the second and f_{IR}, calculated for each of the partner proteins, which is the fraction of interface residues in the first mode that are also in the interface in the second [4]. Since we have two reference monomers $2REB_{core}$ and $3CMW_{core}$ whose side chain conformations may vary at the interface, we used either $2REB_{core}$ or $3CMW_{core}$ as reference depending on the starting monomer in the docking simulation. For more detailed graphical comparisons we also attributed to each residue of the receptor or ligand the best interaction energy of the interface or interfaces in which it was involved in the docking.

Heligeom Analysis

The Heligeom utility `extractHelicalParameters.py`, which couples the ATTRACT output to the screw analysis performed by `heligeom.py`, was used to compute and list the pitch, the number of monomers per turn and the direction of rotation for each docking geometry, together with interaction energy values.

```
python extractHelicalParameters.py docking.att reca.red > screw.txt
```

The results here are redirected to the file `screw.txt`. Selected lines output are shown below.

```
# i    j   N/turn  pitch hand      E
  9  186    4.54   12.35   L   -46.48   #  Z  [1]
 42  113    2.00    0.01   L   -45.00   #  A
 86   95    2.03   52.50   L   -44.78   #  F
 39   21    6.41   90.26   R   -41.14   #  I
 77   85    5.78  106.29   L   -40.21   #  G
 20  204    5.80   72.77   R   -39.41   #  H
 99  117    5.05    0.28   R   -33.91   #  C
 49  129    6.01    0.09   R   -30.69   #  D
215  131   18.04    1.78   R   -30.16   #  E
241   17    3.07    0.01   L   -22.68   #  B
```

Alternatively, an automatic filtering/adjustment post-processing script can be run in order to extract the docking results corresponding to the "Filament" or "Cyclic" categories defined in Figs. S1 and S2 and to filter them as described in Material and Methods ("Processing and filtering the sampling results", main manuscript). The command and its output are similar to the above description of the generic extraction process

```
python extractAndFilter.py docking.att reca.red > filtered_screw.txt
```

From the files `screw.txt` or `filtered_screw.txt`, it is possible to select particular geometries, using ranges of values for the pitch and the number of monomers per turn as selection criteria. For example, the Heligeom command

```
python filterHelicalParameters.py screw.txt -p 70 95 -n 5.5 6.5 -d R
```

accepts screw transformations leading to right-handed (`-d` flag) helices with pitch values between 70 and 95 Å (`-p` flag) and comprising between 5.5 and 6.5 monomers per turn (`-n` flag). The output is here

```
 39   21   6.41   90.26   R   -41.14
 20  204   5.80   72.77   R   -39.41
```

In the same way, geometries consistent with hexameric ring arrangements can be selected using

```
python filterHelicalParameters.py screw.txt -p 0 0.1 -n 5.9 6.1
```

Note that a range of values was indicated in these commands in order to allow for flexibility in the ring closure conditions and the desired overall pitch.

Finally, we used Heligeom to build one or more turns of ring/helix specified by the rotation and translation indices in the `docking.att` output file. For example, the commands

```
python extractHelicalModel.py docking.att reca.pdb 49 129      >  D.pdb
python extractHelicalModel.py docking.att reca.pdb 39  21  2   >  I.pdb
```

were used to build a ring and a helical fiber, respectively. In the first command, the docking result corresponding to translation index 49 and rotation index 129, which we labeled `D` in the above output, was used. This geometry corresponds to an hexameric ring (6.01 monomers/turn, with a nearly vanishing pitch of 0.09 Å). In the second command, the result labeled `I` is a helix with 6.4 monomers per turn and a 90.3 Å pitch. In the latter command, the desired number of helical turns to be output (here 2) was indicated at the end of the command line. Cyclic or helical fibers shown in Figure 2 (main article) were constructed for the `A-I` binding modes using that same procedure.

Figure S5 (Supporting information) displays the same oligomers with the regions corresponding to the extremities of pruned flexible regions represented as color patches. It can be verified that in all cases, these regions are situated outside the monomer-monomer interfaces, which means that the oligomeric form can accommodate the flexible regions. We also represented in orange the amino-acids of the rigid core that participate in binding the N-terminal helix (1-23) in both the 2REB and 3CMW crystal structures. Although the helix does not necessarily bind to that region, the conservation of the helix binding region between the two known RecA structures indicates that it strongly stabilizes the association. In all cases except binding form B (the RecA cyclic trimer), this conserved region was found accessible to the helix binding.

Running times

A typical PTools/ATTRACT docking run on RecA monomers took 7 hours on a single Intel Core 2 Duo running at 3 GHz, or a few minutes when the run was distributed on tens of processors (since PTools facilitates breaking the docking into independent jobs, the speed-up is essentially equal to the number of processors).

Automatic extraction of screw parameters from a PTools/ATTRACT docking output file (about 50,000 poses) took about 5 minutes on a 3 GHz Intel Core 2 Duo processor.

The automatic filtering and cyclic adjustment procedure for the entire set of RecA docking poses took approximately 3 hours on a single 3GHz Intel CPU and retained 9% of the initial 90,489 ATTRACT-generated poses.

Finally, a typical (non optimized) MC run of 10^5 steps on a 3 Gz Intel CPU took 5.5 hours. Introduction of energy cutoffs associated to the restrictions on f_{NAT} value during the MC runs should enable substantial reduction of this duration.

References

[1] Story RM, Weber IT and Steitz TA (1992) The structure of the E. coli RecA protein monomer and polymer. Nature 355, 318-325.

[2] Chen Z, Yang H and Pavletich N P (2008) Mechanism of homologous recombination from the RecA-ssDNA/dsDNA structures. Nature 453, 489-484.

[3] Bastard K, Prévost C, Zacharias M (2006) Accounting for loop flexibility during protein-protein docking. Proteins 62, 956-969.

[4] Méndez R, Leplae R, De Maria L, Wodak SJ (2003) Assessment of Blind Predictions of Protein–Protein Interactions: Current Status of Docking Methods. Proteins 52, 51–67
