# Supplementary material for: An Integrative Approach to the Study of Filamentous Oligomeric Assemblies, with Application to RecA
Source: PLoS One. 2015 Mar 18;10(3):e0116414. doi: 10.1371/journal.pone.0116414 (PMC4364692; doi:10.1371/journal.pone.0116414)
Supplement: S3 Protocol — (PDF) [file pone.0116414.s003.pdf]

Protocol S3: Measuring protein filament groove width

Methods

We define the outer R and inside r radii of the protein filament as the maximum and minimum distances of protein atoms to the helix axis, respectively (Figure S8 A, supporting information). First, a line is drawn between the center of mass of a monomer and its projection on the helix axis On this line, reference points are taken every 1 Å between the outer radius R and the mid point between the inner and outer radii, $(R + r)/2$. The set of reference points is translated by half a pitch so that they are positioned approximately at the center of the groove. For each reference point, a new line is drawn parallel to the helix axis. The maximum diameter of the sphere centered on this line is then computed. The groove width is locally defined as the minimum value of the set of diameters computed from the set of reference points. It represents the maximum possible size of a locally inserted sphere. We construct a similar set of reference points at a next step by interpolating the screw movement of the helix to a half degree. Figure S8 B shows a complete set of reference points that were used to measure the groove width of RecA fibers in Results (below). In that way, we obtain a measure of the groove width for each half degree of a complete turn. This data can then be displayed graphically as a simple line plot. This method has been implemented in a script and can be found in the latest release of PTools.

Results

The groove of a protein filament can be defined as the solvent accessible volume between two consecutive helix turns. There are several ways to characterize the groove, either as a helical cavity with measurable dimensions or by focusing on its function within the filament. Indeed, the groove constitutes a privileged binding site for accessory proteins or ligands and its topology regulates the accessibility to the filament core. Here, we define the groove width as the smallest distance permitting a protein or ligand to penetrate inside the helix. Since the groove width presents local variations, we perform the measurement along a whole helix turn. Figure S9 (supporting information) shows the groove width variation for three different RecA helices, the two known forms X (associated to crystal structure 2REB [1]) and X* (from 3CMW [2]) and a chimerical form obtained by alternating the X and X* binding modes, represented in Figure 6C of the main article.

References

[1] Story RM, Weber IT and Steitz TA (1992) The structure of the E. coli RecA protein monomer and polymer. Nature 355, 318-325

[2] Chen Z, Yang H and Pavletich N P (2008) Mechanism of homologous recombination from the RecA-ssDNA/dsDNA structures. Nature 453, 489-484.
