# Supplementary material for: An Integrative Approach to the Study of Filamentous Oligomeric Assemblies, with Application to RecA
Source: PLoS One. 2015 Mar 18;10(3):e0116414. doi: 10.1371/journal.pone.0116414 (PMC4364692; doi:10.1371/journal.pone.0116414)
Supplement: S1 Dataset — (PDF) [file pone.0116414.s004.pdf]

Dataset S1: Interface residues in the X and X* modes of association

Table S1-1 lists the interface residues in the X and X* binding geometries, together with the associated conservation index as calculated by Karlin and Broccieri [1]. An overview of the geometric arrangement of conserved residues on the monomer surface is shown in Figure S3 (supporting information).

X						X*					
Upper Interface			Lower Interface			Upper Interface			Lower Interface		
Residue		CI[a]	Residue		CI[a]	Residue		CI[a]	Residue		CI[a]
Ser 172		1.00	Asp 94		1.00	Arg 60		0.97	Gly 66		1.00
Gln 173	+	0.81	His 97	+	0.88	Gln 173	+	0.81	Pro 67		0.79
Arg 176	+	1.00	Ala 98	+	0.92	Arg 176	+	1.00	Glu 68		1.00
Asn 213	+	0.11	Leu 99	+	0.86	Lys 177		0.91	Ser 69		1.00
Ala 214	+	1.00	Asp 100	+	0.94	Ala 179		0.43	Lys 72		1.00
Lys 216	+	1.00	Pro 101		0.52	Gly 180		0.23	Thr 73		1.00
Phe 217	+	1.00	Ile 102		0.00	Lys 183		0.00	Glu 96		0.63
Tyr 218	+	0.92	Cys 116		0.05	Gln 184		0.05	His 97	+	0.88
Arg 222	+	1.00	Gln 118	+	0.96	Asn 213	+	0.11	Ala 98	+	0.92
Lys 245		0.97	Pro 119	+	1.00	Ala 214	+	1.00	Leu 99	+	0.86
Val 247		0.82	Asp 120	+	0.91	Lys 216	+	1.00	Asp 100	+	0.94
Lys 248	+	1.00	Thr 121		0.24	Phe 217	+	1.00	Gln 118	+	0.96
Asn 249	+	0.96	Gly 122		1.00	Tyr 218	+	0.92	Pro 119	+	1.00
Lys 250	+	1.00	Gln 124		0.77	Ser 220		0.91	Asp 120	+	1.91
Pro 254	+	1.00	Leu 149	+	1.00	Arg 222	+	1.00	Ser 145		1.00
Phe 255	+	0.45	Thr 150	+	0.28	Lys 248	+	1.00	Ala 147		1.00
Lys 256		0.46	Glu 154	+	1.00	Asn 249	+	0.96	Ala 148		1.00
Gln 257		0.00	Ile 155	+	0.65	Lys 250	+	1.00	Leu 149	+	1.00
			Glu 156	+	0.74	Ile 251		0;30	Thr 150	+	0.28
						Ala 253		0.39	Glu 154	+	1.00
						Pro 254	+	1.00	Ile 155	+	0.65
						Phe 255	+	0.95	Glu 156	+	0.74
									Gln 194		1.00
									Arg 227		0.68
									Val 237		0.00
									Tyr 264		0.72
	0.90*	0.83		0.85*	0.72		0.90*	0.73		0.85*	0.86

Table S1-1: Interface residues in each of the X and X* modes of association, defined as the residues closer than 7 Å from any residue of the interacting monomer in coarse-grained representation; (+) indicate the residues that are common to the X and X* interfaces. The last line reports the average values of the conservation indices (CI) for each interface and for the intersecting region (*) between interfaces in X or X*. (a) the CI values are reported from reference [1]

References

[1] Karlin S and Broccieri L (1996). Evolutionary conservation of reca genes in relation to protein structure and function. J Bacteriol 178, 1881-1894
