# Supplementary material for: An Integrative Approach to the Study of Filamentous Oligomeric Assemblies, with Application to RecA
Source: PLoS One. 2015 Mar 18;10(3):e0116414. doi: 10.1371/journal.pone.0116414 (PMC4364692; doi:10.1371/journal.pone.0116414)
Supplement: S2 Dataset — (PDF) [file pone.0116414.s005.pdf]

Dataset S2: Influence of RecA flexible/mobile components on its binding modes

In the study presented in the main article, the RecA monomers were truncated to their rigid core to explore whether the rigid core of the interface can determine the putative modes of monomer-monomer association. The results suggested that the flexible regions may modulate the mode of association and/or the preference towards one or another binding mode. Here, we further investigated this idea by exploring whether the presence of the ATP cofactor (for the X* binding mode) or the flexible N-terminal linker (for X) modifies the association landscape characterized by the pitch and the number of monomers per turn in Figure 4, main article. In the 3CMW crystal structure [1], the ATP cofactor is located at the interface between two monomers (represented in purple in Figure S7 A, right, supporting information). In the 2REB crystal [2], the same region of the interface that contacts the ATP in 3CMW is occupied by a fraction of the N-terminal linker that folds upon the monomer it belongs to (residues 30 to 37, orange region in Figure S7 A, left).

We performed targeted docking simulations in the presence of the (30-37) linker segment for the X* form (left) and of ATP for the X form (right), and we compared the results with those obtained in the absence of the segment or ATP. Only the results that fulfill the conditions given in the Methods section of the main article (energy lower than -37 RT, f_{NAT} values greater that 0.5 and interface C_{α}-RMSD lower than 3.5 Å) were selected. The comparison is displayed in Figure S7 B, supporting information, in terms of pitch and number of monomers per turn where the blue crosses, corresponding to the results obtained without the linker (left) or without ATP (right), were used as starting points for the Monte Carlo simulations represented in Figure 4 of the main article. While the linker fragment only slightly modifies the sampled regions of the X interface (Figure S7 B, left) and does not modify the interaction energy (not shown), the presence of ATP confines the sampled region to pitch regions below 100 Å and number of monomers per turn between 6.2 and 6.5 (Figure S7 B, right). The presence of ATP also lowers the energy of the selected members of the X* family by ~ 5 RT, which stabilizes that binding mode with respect to the X binding mode. As seen in Figure 4 of the main article, the two binding modes present equivalent values of the interaction energy in the absence of ATP.

References

[1] Chen Z, Yang H and Pavletich N P (2008) Mechanism of homologous recombination from the RecA-ssDNA/dsDNA structures. Nature 453, 489-484.

[2] Story RM, Weber IT and Steitz TA (1992) The structure of the E. coli RecA protein monomer and polymer. Nature 355, 318-325.
